# Designing a trial of early electrical stimulation to the stroke-affected arm: Qualitative findings on the barriers and facilitators

**DOI:** 10.1177/03080226211008706

**Published:** 2021-09-26

**Authors:** Dawn-Marie Walker, Joanna Fletcher-Smith, Nikola Sprigg, Anand Pandyan

**Affiliations:** 1 School of Health Sciences, Highfield Campus, 7423 University of Southampton, Southampton, UK; 2 Faculty of Medicine and Health Sciences, 6123 University of Nottingham, Nottingham, UK; 3 Faculty of Medicine & Health Sciences, Nottingham City Hospital, Nottingham, UK; 4 School of Health and Rehabilitation, 4212 Keele University, Keele, UK

**Keywords:** Electrical stimulation, stroke, rehabilitation, qualitative, trial design

## Abstract

**Introduction:** This study aimed to explore the barriers and facilitators to implementing early therapeutic electrical stimulation (ES) treatment from both the patient and therapist perspectives as part of a feasibility study.

**Methods:**

Design: Interviews were conducted with patients and their carers and focus groups with the therapists post-intervention period.

Setting: Interviews were in the patient’s homes and for the focus groups in a specialist stroke unit in Nottinghamshire.

Subjects: Fifteen patient participants (34% of sample) were interviewed (intervention *n* = 9; control group *n* = 3; carers *n* = 3). Sixteen therapists (9 occupational therapists; 7 physiotherapists) took part in the three focus groups.

Intervention: Participants were randomized to receive usual care or usual care and ES to wrist flexors and extensors for 30 min, twice a day, 5 days a week for 3 months.

**Findings:** The barriers to ES treatment cited by the therapists outweighed the barriers mentioned by patients. Therapists’ barriers included lack of confidence and staff knowledge regarding ES and time pressures of delivering the ES. No patients mentioned time as a barrier and considered the treatment regime to be acceptable; however, lack of staff support was mentioned 14 times by them.

**Conclusion:** Although initially the perceived barrier for therapists was time restrictions, after analysing the data, it appears that confidence/knowledge is the real barrier, and time is the manifestation of this underlying self-doubt. Patients were able to confidently self-manage treatment, and although efficacy was not measured, patients volunteered information regarding its perceived benefit, and no adverse effects were reported.

## Introduction

### Post-stroke upper limb complications

Stroke continues to be a global leading cause of adult disability (National Audit Office, 2010). Around 70–80% of stroke survivors are affected by upper limb hemiparesis which is a chronic and disabling problem for an estimated 40% (Royal College of Physicians, 2012). In the presence of persistent paresis, arm muscles atrophy rapidly, and patients, particularly those with spasticity and pain, are at an increased risk of developing painful muscle contractures (fixed joint deformities) (Malhotra et al., 2011). Contractures have 6-month incidence rates of 22% (elbow and forearm) and 13% for wrist and hand (Kwah et al., 2012) and can become established as early as 6 weeks after stroke (Malhotra et al., 2011). One possible cause for contracture is the lack of adequate upper limb therapy input (Intercollegiate Stroke Working Party, 2012, 2016; National Institute for Clinical Excellence, 2013). In England, patients do not always receive the recommended 45 min per day therapy (Intercollegiate Stroke Working Party, 2012). Kaur et al. (2012) found that on average, patients on stroke units receive between 0.9 and 7.9 min of upper limb therapy. Therefore, there is a need to increase the amount of therapy targeted specifically at the arm and hand in a cost- and resource-effective manner. One way is to use technology and encourage self-management of treatment by patients and their informal carers. Furthermore, empowerment via self-management has significant positive effects on functional recovery (Sit et al., 2016).

Surface neuromuscular electrical stimulation (ES) is an inexpensive technology that has been developed to stimulate a muscle contraction and provide rehabilitative therapy. For those patients unlikely to make functional gains, prevention of complications such as pain and contractures should be the focus of therapeutic interventions, and there is some evidence that treatment with ES is of potential value (Dimitrijević and Soroker, 1994; Malhotra et al., 2013; Pandyan et al., 1997; Rosewilliam et al., 2012).

Previous pilot trials of ES have only focussed on stimulation of the extensor muscles and have not stimulated the flexor muscle group but have demonstrated some benefits in terms of slowing the rate of deterioration, and in some cases, facilitating recovery; however, effect sizes were small (Malhotra et al., 2013; Pandyan et al., 1997; Rosewilliam et al., 2012). These studies concluded that the treatment was not given for long enough, and premature discontinuation of therapy may have reduced any potential therapeutic effect. However, the muscles at risk of shortening are the wrist and forearm flexors, and the most effective method of loading the soft tissue structures of the flexors in patients who are unable to fully activate their muscles is by electrically stimulating these muscles. Furthermore, evidence suggests that early initiation (24 h post-stroke) of rehabilitation interventions and high intensity of treatment can enhance the chances of neurological recovery (Bernhardt et al., 2013, 2017; Biernaskie et al., 2004; Kwakkel et al., 2003, 2006). Previous studies do not recruit or prescribe ES early after stroke, for example, an underpowered trial found no effect from ES; however, they recruited up to 6 months post-stroke (Wilson et al., 2016). This is borne out in meta-analyses which ascertained that ES significantly improved Activities of Daily Living when initiated within 2 months; however, this effect disappeared when initiated by 6 months (Eraifej et al., 2017).

### The early electrical stimulation to prevent complications in the arm post-stroke study

Early electrical stimulation to prevent complications in the arm post-stroke (ESCAPS) evaluated the feasibility of delivering a definitive randomized controlled trial of usual care versus usual care and early ES therapy to the wrist extensor and wrist flexor muscles to prevent the post-stroke complications of pain and contractures in the arm (Fletcher-Smith et al., 2016). The full protocol and methods are reported elsewhere (Fletcher-Smith et al., 2016, 2019), but briefly ESCAPS recruited 40 patients admitted to one stroke unit in England within 72 h post-stroke. They were randomized 1:1 to receive either the intervention, that is, ES,^1^ for 30 min, twice a day, Monday to Friday for 3 months in conjunction with usual care, or the control group of usual care. Participants randomized to the control group did not receive ES therapy but received all usual care which included all the therapy interventions that are standard practice as per national clinical guidelines (Intercollegiate Stroke Working Party, 2016). It was not possible to collect data on the nature or volume of usual care. Intervention participants were provided with a device, so they could continue when discharged.

Participants on the trial were assessed on a series of outcome measures at baseline (prior to randomization), 3 months (immediately following the 3-month intervention phase), 6 months and 12 months. The publication of the quantitative outcomes is published elsewhere (Fletcher-Smith et al., 2019); here, we report the findings of the supplementary qualitative study which explored the barriers and facilitators to implementing early therapeutic ES treatment from both the patient and therapist perspectives. Patient, carer and therapists’ opinions were sought on both treatment acceptability and aspects of the trial design for the purpose of informing protocol development for a subsequent definitive trial.

## Methods

Ethical approval [REC reference: 15/EM/0006] was granted for a sample of patients/carers (*n* = 12) and therapists (*n* = 16) involved in the ESCAPS feasibility RCT to be recruited to the qualitative study.

### Patient and carer interviews

The information sheet provided to the participants prior to recruitment to the feasibility study stated that they may be invited to participate in the interviews. A representative sample based on age, sex and baseline National Institutes of Health Stroke Scale scores of participants was recruited for the interviews. Participants who had completed their final follow-up assessment and who would be able to communicate freely were invited. After giving informed written consent, participants and their informal carers were interviewed in their own homes by Kate Allatt (KA) and JFS. The patients all knew JFS as she had carried out all of the follow-up assessments and most of the baseline assessments. Kate Allatt was not known to them prior to the interview. Arguably, the prior relationship between JFS and the participants may have biased the data; however, the questions were trial focussed rather than efficacy, and upon analyses, there were no qualitative differences between the data obtained between the two interviewers.

Interviews lasted between 30 and 60 min and were audio recorded and transcribed verbatim. The researcher followed an ethically approved semi-structured interview guide. Intervention participants were asked questions on the following topics:

1. How they would describe the ES treatment.
2. Their experience of the first treatment session.
3. Their views on the early application (within 72 h post-stroke) of ES treatment.
4. The sensation of the treatment.
5. The perceived effect of the treatment.
6. The prescribed wearing schedule of the device (frequency of treatments, duration and dose).
7. Their ability to use the device independently.
8. Any problems experienced in using the device.
9. Their experience of being involved in the clinical trial.

Furthermore, participants from the control group were interviewed and asked about their experience of being involved in a trial where they were randomized to usual care.

### Therapist focus groups

All of the National Health Service (NHS) therapists (physiotherapists and occupational therapists) who had been involved in supporting the delivery of the ES treatment as part of the ESCAPS trial were invited to participate in a focus group. Three focus groups were held during the intervention phase. Each was held in a meeting room on the hospital grounds and lasted 1 h. Two researchers who were known to the therapists (JFS and KA) led the focus groups. Once again bias was minimized as the questions, guided by an ethically approved topic guide, were trial focussed rather than perceptions of efficacy.

Focus group participants were asked to discuss the following topics:

1. Views on the patient inclusion/exclusion criteria for the ESCAPS study.
2. Assessments used in the study.
3. Their experience of administering the ES treatment.
4. Experience of training patients and carers to apply the treatment.
5. Factors that might facilitate implementation of this type of intervention in clinical practice.
6. Possible barriers that might affect implementation of the intervention.
7. Experience of delivering ES as part of a randomized controlled trial.

The focus groups were audio recorded and transcribed verbatim.

### Analyses

After verbatim transcription, the transcripts were read a number of times by two researchers JFS and DMW after checking the transcripts whilst listening to the audiotapes to ensure that they were accurate and to add any contextual or reflective data. Framework analysis was used (Ritchie and Spencer, 1994). After familiarization, both researchers independently began coding the data. After a few transcripts were coded, the researchers met to compare the codes and their groupings to agree on a set of codes going forward, thereby developing an analytical framework. After the framework had been determined, this was used for the analyses of the following transcripts. Both researchers worked together to chart the data from the transcripts onto the framework matrix before initial interpretation. The framework matrix and interpretation was then discussed in the trial management meetings with the other collaborators including an expert by experience.

## Findings

Fifteen patient participants representing 34% of the sample were interviewed including intervention (*n* = 9), control group (*n* = 3) and carers (*n* = 3) involved in supporting the treatment. Carer’s demographic data were not collected; however, the demographic details of the patient participants interviewed are included in Table 1.

**Table 1.** Demographic details of patients interviewed.

Gender Female *N* (%)	Age Mean Min–max	Stroke type Infarct *N* (%)	Side of stroke Left: right	Baseline NIHSS Mean Min–max	Baseline BI^a^ score Mean Min–max	Baseline ARAT^b^ Mean Min–max
7 (58%)	74 years 62–84 years	10 (83%)	6: 6	9 2–24	7.4 0–20	10 0–51

^a^BI: Barthel ADL index.

^b^ARAT: action research arm test.

Sixteen female therapists (9 occupational therapists and 7 physiotherapists), NHS bands 3–6 (occupational/physiotherapist assistants *n* = 13) and band 7 (qualified occupational/physiotherapists *n* = 2) to band 8 (clinical leads *n* = 1) participated in the three therapist focus groups. These bands which are underpinned by a Knowledge and Skills Framework are designed to ensure equitable pay and opportunities for progression for all non-medical staff. The bands are based on qualifications and years of experience. This sample included staff who had been involved in establishing the correct area for electrode placement and providing the initial treatment (qualified band 5 and above staff) or in supporting subsequent treatment sessions (band 3 and above).

The data from the patients, carers and the therapy staff formed overarching themes related to the clinical application of early ES therapy and issues related to the trial design and delivery.

### Confidence and knowledge

The barriers to ES treatment cited by the therapists outweighed the barriers mentioned by patients. Therapists’ barriers included lack of confidence and staff knowledge (mentioned 29 times):

*‘Probably us OTs are less comfortable with using electrical stimulation than physios are because they would use TENS gloves and things like that so we are probably less comfortable with it and it would take a bit more practice’* [Occupational Therapist, focus group 2].

*‘You don’t know whether you’ve got it in the right place. We’re shown how to do it and yes we know our anatomy, or we get the book out and look at it, but you just think, you just doubt yourself don’t you, or if you’re doing it right or not?’* [Physiotherapist, focus group 3].

*‘It was difficult to get movement with this particular patient that we had to go back to the text book and still didn’t know very well’* [Occupational Therapist, focus group 3].

Due to these issues, therapists wanted more training and support and also suggested a troubleshoot list/list of frequently asked questions would be useful.

Some of the confidence issues were due to pre-conceived ideas about the importance of ‘normal movement’. Therapists were concerned about the positioning of the arm and hand for treatment (mentioned in two out of three focus group discussions) and were concerned with eliciting abnormal movement as a result of the ES treatment:

*‘There’s been times when there’s been multiple therapists going in and trying to get the right and the perfect position’* [Physiotherapist, focus group 3].

*‘I don’t want to be feeding the wrong pattern of movement for 30 minutes twice a day’* [Physiotherapist, focus group 3].

*‘I think there will be confidence issues and you know, is this working? Am I doing this in the right way? And if you don’t get that visual kind of payback that it is working and you’ve got the perfect wrist extension. I think we all want the perfect movement and I know actually, the project is you don’t have to have the perfect movement but I think that we all kind of aim to achieve a nice wrist extension that looks good and we don’t have a deviation. A nice flexion that looks good. And I guess that’s how we are all taught, especially if it’s a band 5, it’s like normal movement, normal movement, so that when we get non normal movement we don’t like it very much’* [Physiotherapist, focus group 3].

Comments also suggested a perceived complexity of the intervention by therapy staff:

*‘I don’t have enough experience with that equipment or how it’s working because I don’t seem to ever be able to do it right every time I try. I never seem to be able to get it right’* [Occupational Therapist, focus group 3].

On the contrary, all patient participants who had been allocated to the intervention arm of the trial reported being able to confidently self-manage the ES treatment (mentioned 19 times):

*‘Actually it wasn’t difficult, I mean I didn’t struggle with it at all’* [Participant 1, female, aged 70].

*‘I was quite happy to start straight away and I was able to put the electrodes on myself and do it so I didn’t have any problems there. Yes, I just got on with it…I just carried on using it. It wasn’t difficult’* [Participant 2, female, aged 67].

These opposing opinions could be due to dialectic model of reasoning (Edwards and Jones, 2007). Underlying the therapist’s approach is hypothetico-deductive reasoning which underpins the biomedical model of health care (Elstein et al., 2013). Therapists quantify, measure and grade what they are seeing against their held ‘truth’ which is what they expect. However, underlying the patient perspective is the self-determination theory which is argued to underpin home exercise regimen with the most adherent being those with intrinsic motivation, for example, believing that completing the treatment will benefit them (Deci and Ryan, 2000, 2010). Although efficacy was not questioned during the interviews, many patients volunteered this information and perceived the ES to be of benefit (mentioned 27 times by 7 patients).

### Time

Another barrier was the perceived time pressures that were cited by therapists during the focus group discussions (mentioned 11 times):

*‘I reckon one of the biggest things would just be the time factor. The feasibility of making sure it’s on twice a day because on some days on any ward it’s just so busy that it might just get missed’* [Physiotherapist, focus group 2].

*‘If I might have ten new patients that day, I’ve not only got to do the new patients but then I’ve got to do that as well and don’t have that amount of time really’* [Physiotherapist, focus group 3].

*‘Especially when it’s Friday afternoon and they want them to go for the weekend, the last thing you’re going to have time to do on Friday afternoon is teach the family how to use electrical stimulation’* [Occupational Therapist, focus group 3].

No patients mentioned time as a barrier and considered the treatment regime to be acceptable (mentioned 17 times by 11 patients). However, lack of staff support was mentioned 14 times during the patient and carer interviews:

*‘The therapists didn’t really show me how to use it because I think they were in a mess themselves sometimes, you know, but it was alright’* [Participant 4, female patient aged 80].

*‘The problem was I didn’t know how to put it on in the hospital. Some nurses did. Then there were certain people that we could if they were on that shift. We would say, can you help me, but if they were busy they couldn’t you see. It was whether they were busy or not. On the stroke ward I think sometimes if they hadn’t got the staff or if they were busy it got overlooked you know’* [Participant 6, female patient aged 66].

In terms of the timing of the early intervention, it was viewed as a barrier by therapists (mentioned during two focus groups) because of the pressure it placed on staff but was considered acceptable to patients (12 mentions during eight of the patient interviews).

## Discussion

The data demonstrated a discord between the perceptions of the clinicians and the patients, with both overarching themes of confidence and knowledge, and time being perceived as a barrier by clinicians, whilst the patients did not perceive them as barriers. Confidence/knowledge was the main barrier perceived by clinicians, and time appeared to be a manifestation of this underlying self-doubt. Patients however argued that they were able to confidently self-manage treatment. They also argued that the treatment regimen was acceptable. However, they did report lack of staff support which arguably could be due to either time pressures or confidence and knowledge of staff.

These themes resonate with previous research. One qualitative study looked at the perspectives regarding assistive technologies for the treatment of stroke in patients, caregivers and health professionals. They found that patients were keen to self-manage and argued that if they were taught how to use the devices in hospital, they would be happy to continue at home. However, once again the health professionals (all but one being physiotherapists or occupational therapists) were more ambivalent about using assistive technologies, citing the time required to prescribe and teach people how to use them, as well as preparing, setting up and maintaining the assistive technologies as an issue (Demain et al., 2013). Patients in Demain et al.’s study noted that therapists were overworked which echo the participant’s perspectives in the current study.

Another study which collected data from 298 physiotherapists regarding using functional ES found that time was mentioned as a barrier to usage more often than any other resource. Therapists reported short treatment sessions and lengths of stay, large caseloads and the time required to set up the units and change their parameters as barriers (Auchstaetter et al., 2016). Underpinning this is that healthcare models have been developed by service providers, who prioritize different outcomes from patients (Hammel et al., 2013; Ripat and Booth, 2005), which results in a bias towards independence and safety, rather than changes in factors which are meaningful to patients such as quality of life (Steel, et al., 2016).

Knowledge has also been argued as a barrier to using assistive technologies by earlier research. Demain et al. (2013) found that health professionals were concerned about a lack of efficacy research and were worried about the potential risk of harm. Lack of knowledge was also reported by their patient participants who argued that their therapists perhaps lacked the knowledge about technologies. Demain and team found that therapists were extremely risk adverse, whereas the patients and carers were more willing to accept risks. This is supported by a more recent study (Auchstaetter et al., 2016). Correlations were found between using functional ES and knowledge of the evidence (*r* = 0.279, *p* < 0.001) and education in its use (*r* = 0.15, *p* = 0.013). Therapists acknowledged the importance of hands-on experience and ultimately report that a facilitator for using functional ES was the therapist feeling comfortable and confident in applying it. These differences in perspectives between patients and therapists limit the effectiveness of patient-centred approaches (Steel et al., 2016).

The findings from the patient and carer interviews and the therapist focus groups informed a number of improvements to the ongoing feasibility study and the protocol for the subsequent definitive trial. For example, in addition to the therapist training workshops, a number of staff drop-in sessions were provided for the nursing staff on the stroke unit to provide them with a clear and simple explanation of the purpose of the ESCAPS study, the aims of the ES treatment, and how to apply the treatment, remove the electrode pads from the skin and correctly store the electrodes when not in use. Therapists were also encouraged to attend any of these drop-in sessions for further refresher training if required. Although efficacy was not measured, patients volunteered information regarding their perceived benefit, and no adverse effects were reported.

## Conclusion

This qualitative work will inform the design of a definitive trial. Ultimately, ES may provide a cost-effective solution to achieving recommended therapeutic targets which may result in real patient benefit.

## Key Points for Occupational Therapy

1. A clinical trial in early ES for stoke-affected upper limbs is feasible.
2. Therapists’ barriers included lack of knowledge regarding ES and time pressures of delivering the ES.
3. Patients considered the treatment regime to be acceptable; however, lack of staff support was mentioned.

## Note

1 The NeuroTrac Rehab Verity Medical Limited in compliance with the European Union Medical Device Directive MDD93/42/EEC under the supervision of SGS, notified body number 0120 CE mark status. Verity Medical Ltd. is certified by SGS to the following quality standards: ISO 9001:2008, ISO13485:2003.
